# How Spiders Spin Silk

**DOI:** 10.1371/journal.pbio.1001922

**Published:** 2014-08-05

**Authors:** Robin Meadows

**Affiliations:** Freelance Science Writer, Fairfield, California, United States of America

Spider silk is wonderful stuff—light as the breeze and stretchy yet stronger than steel. People can manufacture synthetic fibers, such as Kevlar, that come close but can't begin to match the process spiders use. Their silk proteins, called spidroins, rapidly convert from the soluble form to solid fibers at ambient temperatures and with water as the solvent. Not only is this beyond us, we don't even know how spiders do it. Now, in this issue of *PLOS Biology*, new research by Anna Rising, Jan Johansson, and colleagues shows that silk formation involves structural shifts at either end of the spidroin and that these shifts are completely different, overturning the hypothesis that these protein terminals play similar roles.

Spidroins are big proteins of up to 3,500 amino acids that contain mostly repetitive sequences, and the nonrepetitive N- and C-terminal domains at opposite ends are thought to regulate conversion to silk. These terminal domains are unique to spider silk and are highly conserved among spiders.

Spidroins have a helical and unordered structure when stored as soluble proteins in silk glands, but, when converted to silk, they contain β-sheets that lend mechanical stability. We know that there is a pH gradient across the spider silk gland, which narrows from a tail to a sac to a slender duct, and that silk forms at a precise site in the duct. However, further details of spider silk production have been elusive.

To discover the basis for the pH gradient in the silk gland, the researchers studied the orb weaver spider *Nephila clavipes*, which reaches 7.6 centimeters wide and spins webs up to 3 meters across. Poking ion-selective microelectrodes into the silk gland revealed that the pH fell from 7.6 to 5.7 between the base of the tail and halfway down the duct, which was as far as the electrodes fit. This pH gradient is much steeper than previously thought.

The microelectrodes also showed that bicarbonate ions rise between the base of the tail and the far end of the sac. Taken together, these ion patterns suggest that the pH gradient is due to carbonic anhydrase, an enzyme that converts carbon dioxide and water to bicarbonate and hydrogen ions. In support of this idea, a carbonic anhydrase inhibitor called methazolamide collapsed the pH gradient.

**Figure 1:**
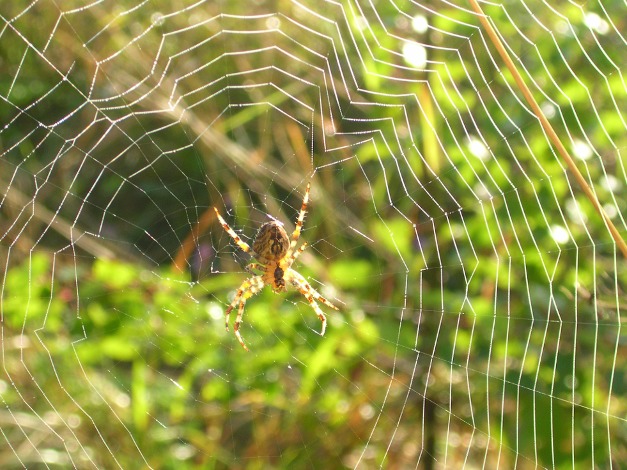
An *Araneus* spider in its orb web. *Image credit: Anna Rising.*

Next, the researchers investigated how the pH range across the silk gland affects silk proteins, using N- and C-terminal domains isolated from the spidroins of another orb weaver, *Araneus ventricosus* (Figure 1). They found that both domains undergo structural changes at the pH found in the duct. Importantly, this is also where carbonic anhydrase activity is concentrated.

The researchers also found pH had opposite effects on the two domains' stability to temperature and urea, which was a surprise given that the domains had been suggested to have a similar impact on silk formation. The N-terminal dimerized at pH 6—which is found in the beginning of the duct—and became increasingly stable as the pH dropped along the duct. In contrast, the C-terminal domain destabilized as the pH dropped, gradually unfolding until it formed the β-sheets characteristic of silk at pH 5.5.

What makes the C-terminal domain unfold? This end of the spidroin has a salt bridge joining two helices, and mutations that interfere with this bridge also destabilize the C-terminal domain. Underscoring its importance, the salt bridge is conserved in spidroins from several spider species. The researchers believe that protonating amino acids in the salt bridge would also destabilize the C-terminal domain, facilitating conversion of spidroins from helices to β-sheet fibrils.

These findings led the researchers to propose a new “lock and trigger” model for spider silk formation. Gradual dimerization of the N-terminal domains could lock spidroins into multimers, while the β-sheet fibrils at the C-terminals could serve as nuclei that trigger rapid polymerization of spidroins into fibers. Interestingly, the C-terminal β-sheets are similar to those in the amyloid fibrils characteristic of diseases such as Alzheimer disease.

This work brings us closer to unraveling the mystery of spider silk, explaining how it can form so quickly—faster than a meter per second—as well as how its formation can be confined to the spinning duct. Moreover, because the N- and C-terminal domains of spidroins are found nowhere else, this lock and trigger formation is likely unique to spider silk. Besides being essential to producing biomimetic spidroin fibers, knowing how spiders spin silk could give insights into natural ways of hindering the amyloid fibrils associated with disease.

**Andersson M, Chen G, Otikovs M, Landreh M, Nordling K, et al. (2014) Carbonic Anhydrase Generates CO_2_ and H^+^ That Drive Spider Silk Formation Via Opposite Effects on the Terminal Domains.** doi:10.1371/journal.pbio.1001921

